# Eyelid Arteriovenous Malformation With Orbital Fistula: Literature Review and Case Report

**DOI:** 10.7759/cureus.97349

**Published:** 2025-11-20

**Authors:** Inês Ludovico, Nuno Rodrigues Alves, Tiago Neves, Isabel Fragata, Ana Magriço

**Affiliations:** 1 Ophthalmology, Unidade Local de Saúde São José, Lisbon, PRT; 2 Neuroradiology, Unidade Local de Saúde São José, Lisbon, PRT

**Keywords:** arteriovenous malformations, eyelid mass, oculoplastic surgery, orbital fistula, vascular embolization

## Abstract

Orbital arteriovenous malformations (AVMs) are rare entities, either congenital or acquired, that can significantly impair ocular function and aesthetics. Diagnostic evaluation requires multimodal imaging, and treatment is extremely complex, not only due to the limited information available in the literature, but also because of the high recurrence rate of the lesion, therefore requiring a multidisciplinary approach. Treatment involves a multimodal approach, often requiring AVM embolization followed by surgical excision. Endovascular embolization with agents such as ethylene-vinyl alcohol (Onyx®) or N-hexyl cyanoacrylate (Magic Glue®) should be ideally performed prior to subsequent surgical approaches. Recurrence is frequent, especially when nidus occlusion is incomplete, underscoring the importance of long-term follow-up and reintervention when necessary. We present a review of the recent literature and a clinical case of a left upper eyelid AVM associated with an orbital fistula in a young patient, managed with endovascular embolization and surgical excision.

## Introduction

Orbital AVMs are high-flow vascular lesions characterized by an abnormal network of direct communications between arteries and veins, without normal capillary interposition [1]. Although they can be acquired, they are often congenital and become clinically evident in young adulthood [2,3].

Palpebral AVMs are particularly rare and, when associated with an orbital fistula, may cause significant ocular and aesthetic manifestations such as ptosis, pulsatile mass, and risk of hemorrhage [4,5]. Diagnostic evaluation requires multimodal imaging, and treatment is extremely complex, not only due to the limited information available in the literature but also because of the high recurrence rate of the lesion, therefore requiring a multidisciplinary approach. Digital subtraction cerebral angiography (DSA) remains the gold standard for evaluating these lesions, allowing mapping of the nidus, feeding arteries, and venous drainage pathways [6]. Orbital Computed Tomography (CT) also provides greater detail of the bony structures, enabling good identification of calcifications and bone involvement, which is useful for surgical planning [7]. Magnetic Resonance Imaging (MRI) offers better soft tissue contrast and detailed delineation of the AVM structure, including flow characteristics and extension into adjacent tissues [8].

Treatment involves a multimodal approach, often requiring AVM embolization followed by surgical excision. Endovascular embolization with agents such as ethylene-vinyl alcohol (Onyx®) or N-hexyl cyanoacrylate (Magic Glue®) has shown efficacy in reducing lesion flow and volume, facilitating subsequent surgical approaches [9]. Recurrence is frequent, especially when nidus occlusion is incomplete, underscoring the importance of long-term follow-up and reintervention when necessary [10].

## Case presentation

**Figure 1:**
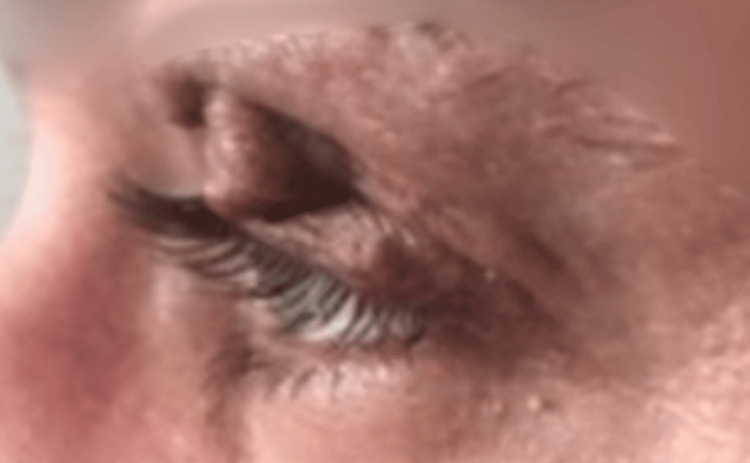
Left upper eyelid pulsatile, violaceous mass measuring 6×6 mm, without ulceration or pigmentation

A 35-year-old male patient with a history of congenital AVM was referred to our center with complaints of a progressively enlarging violaceous mass on the left upper eyelid. He denied any history of trauma or previous hemorrhagic episodes. On examination, one could find a pulsatile, violaceous mass measuring 6×6 mm, without ulceration or pigmentation (Figure 1).

There was evident ptosis, with a palpebral fissure height of 6 mm and a margin reflex distance 1 (MRD1) of 1 mm, while superior levator palpebrae function was within normal range (15 mm). Best-corrected visual acuity (BCVA) was 20/20, bilaterally, with normal biomicroscopy and fundus examination. Intraocular pressure was of 12 mmHg bilaterally, with no signs of ocular venous congestion.

**Figure 2:**
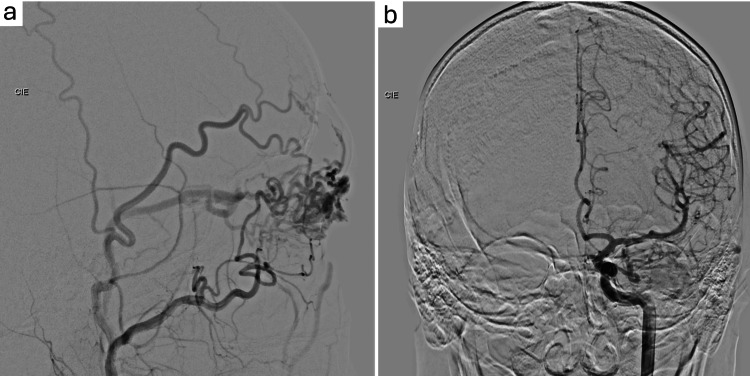
Intraorbital arteriovenous malformation (AVM) in the left upper eyelid region measuring 17.5 mm at its largest diameter a: Intraorbital AVM in the left upper eyelid (Lateral View); b: Intraorbital AVM in the left upper eyelid (Frontal View).

DSA revealed a left intraorbital nidal AVM in the superior eyelid region measuring 17.5 mm at its largest diameter, supplied by the ophthalmic, superficial temporal, and anterior deep temporal arteries (branch of the internal maxillary artery). Venous drainage was predominantly via the facial vein and cavernous sinus (Figure 2).

Under the interventional neuroradiology approach, after administration of 8 mg of dexamethasone and 4000 U of heparin, embolization of the sphenoidal branch of the internal maxillary artery was performed using ethylene-vinyl alcohol (Onyx®), excluding the inferior component of the nidus. The distal branch of the ophthalmic artery was also embolized, resulting in a significant reduction of vascular flow.

**Figure 3:**
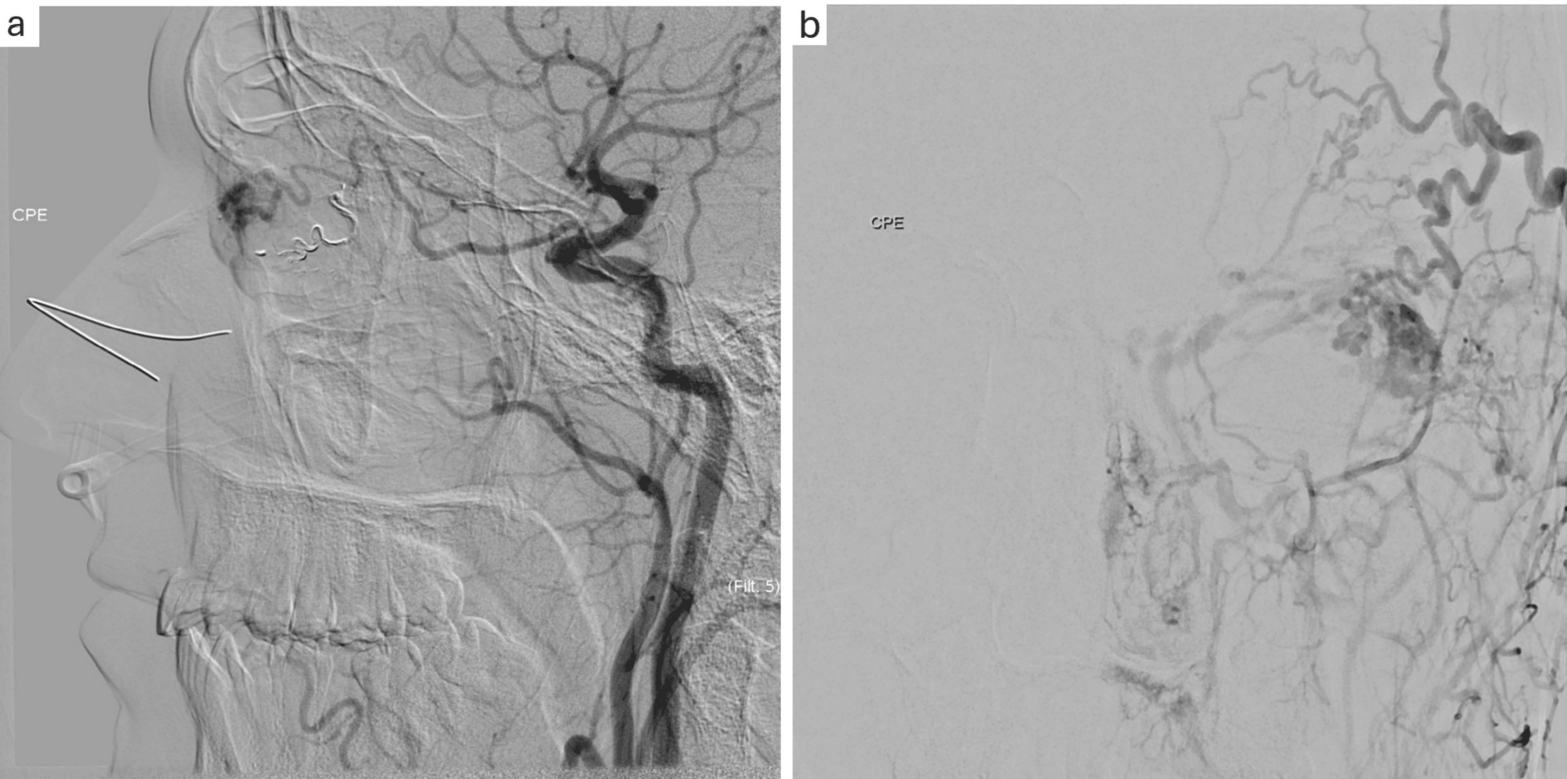
Intraorbital arteriovenous malformation (AVM) after embolization procedure showing marked reduction of lesion size with minimal residual flow a: Intraorbital AVM after embolization with minimal residual flow (Lateral View); b: Intraorbital AVM after embolization with minimal residual flow (Frontal View).

Two weeks after the first procedure, control angiography revealed a residual AVM, and additional embolizations were carried out in two branches of the superficial temporal artery and two branches of the ophthalmic artery using N-hexyl cyanoacrylate (Magic Glue®), achieving marked reduction with minimal residual flow (Figure 3).

Surgical excision of the previously embolized vessels was then performed under local anesthesia. The approach consisted of an incision in the middle third of the eyelid, with subcutaneous dissection to the base of the lesion and cauterization of remaining arterial branches.

**Figure 4:**
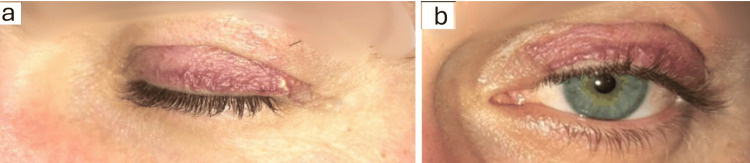
Two months postoperatively, the left upper eyelid showing complete wound healing with palpebral telangiectasias a: Palpebral telangiectasias (Lateral View); b: Palpebral telangiectasias (Frontal View).

Two months after surgery, complete wound healing was observed; however, there was partial recurrence manifested by palpebral telangiectasias (Figure 4).

A second surgical procedure was performed in order to excise the residual telangiectatic tissue. Final post-operative palpebral fissure height was of 9 mm and MRD1 of 4 mm, with preserved superior levator palpebrae function. The patient remains under follow-up at our center and so far no recurrence has occurred. 

## Discussion

Orbital and periorbital AVMs represent a diagnostic and therapeutic challenge due to their rarity and anatomical complexity. The case presented here highlights the importance of a structured, multidisciplinary, and sequential approach. Differential diagnosis of vascular eyelid masses includes capillary hemangiomas, orbital varices, lymphangiomas, venous or mixed AVMs, and indirect carotid-cavernous fistulas. Complementary diagnostic modalities are essential for differentiation and therapeutic planning. DSA remains the first choice for complete mapping of the nidus, identification of feeding arteries, and venous drainage pathways. It is essential for guiding selective embolization and assessing therapeutic efficacy [6]. CT is useful for assessing the extent of the lesion, orbital bone erosions, and its impact on adjacent structures [7]. MRI provides superior soft tissue contrast and detailed delineation of the AVM structure, including flow characteristics and extension into adjacent tissues [8].

The treatment of orbital AVMs depends on the clinical presentation, anatomical location, number of feeding arteries, and risk of complications. The ideal therapeutic strategy aims to control symptoms, reduce lesion flow, and minimize recurrence. The main embolic agents include: 1) Ethylene-vinyl alcohol (Onyx®) - The main advantages of this agent are its non-adhesiveness, progressive solidification, cohesiveness, high vascular penetration, and minimal inflammatory effect on the endothelium. It penetrates deeply, embolizing the feeding vessels and inducing complete devascularization of the nidus, as well as of the afferent and efferent branches [11]; 2) N-hexyl cyanoacrylate (Magic Glue®) - This is a rapidly polymerizing monomer with an occlusive efficacy of 87%, though it carries a higher risk of delayed displacement of the embolic agent [12]; and 3) Precipitating Hydrophobic Injectable Liquid (PHIL) - It exhibits an anterograde columnar embolic flow, allowing an occlusive efficacy of 77% [13].

Surgery is indicated for lesions that are localized and accessible, particularly following embolization to reduce the risk of bleeding. The sequential combination of embolization and surgery is the preferred approach for complex orbital AVMs. Studies such as those by Kim et al. [2] and Gemmete et al. [10] demonstrate higher success rates with this approach, along with lower recurrence rates.

Experimental studies have explored the use of anti-angiogenic agents (such as bevacizumab) and vascular growth modulators in difficult-to-control AVMs. Although promising, these approaches still lack robust evidence [14].

## Conclusions

Palpebral orbital AVMs are rare lesions that require early diagnosis and a multidisciplinary approach for effective control. The combination of selective embolization and surgery allows satisfactory outcomes. The prognosis depends on the extent of the lesion, the success of nidus occlusion, and the number of feeding arteries. Orbital AVMs carry a significant risk of recurrence, particularly when nidus embolization is incomplete. Continuous clinical and imaging follow-up is essential, and additional treatment may be required in cases of recurrence.
